# Intestinal Intervention Strategy Targeting Myeloid Cells to Improve Hepatic Immunity during Hepatocarcinoma Development

**DOI:** 10.3390/biomedicines9111633

**Published:** 2021-11-06

**Authors:** Adrián Bouzas Muñoz, Juan Antonio Giménez-Bastida, Aurora García Tejedor, Claudia Monika Haros, Marta Gómez de Cedrón, Ana Ramírez de Molina, José Moisés Laparra Llopis

**Affiliations:** 1 Molecular Immunonutrition Group, Madrid Institute for Advanced Studies in Food (IMDEA Food), Ctra Cantoblanco 8, 28049 Madrid, Spain; adrian.bouzas@imdea.org; 2 Molecular Oncology Group, Madrid Institute for Advanced Studies in Food (IMDEA Food), 28049 Madrid, Spain; marta.gomezdecedron@imdea.org (M.G.d.C.); ana.ramirez@imdea.org (A.R.d.M.); 3 Canaan Research and Investment, 28003 Madrid, Spain; 4 Laboratory of Food and Health, Research Group on Quality, Safety and Bioactivity of Plant Foods, Department Food Science and Technology, Campus de Espinardo, CEBAS-CSIC, 30100 Murcia, Spain; jgbastida@cebas.csic.es; 5 Faculty of Health Sciences, Valencian International University (VIU), Pintor Sorolla 21, 46002 Valencia, Spain; agarciate@universidadviu.com; 6 Instituto de Agroquímica y Tecnología de Alimentos (IATA), Consejo Superior de Investigaciones Científicas (CSIC), Av. Agustín Escardino 7, Parque Científico, Paterna, 46980 Valencia, Spain; cmharos@iata.csic.es

**Keywords:** macrophages, lipid homeostasis, immunonutrition, hepatocarcinoma

## Abstract

Innate immunity in the tumor microenvironment plays a pivotal role in hepatocarcinoma (HCC) progression. Plant seeds provide serine-type protease inhibitors (SETIs), which can have a significant influence on liver inflammation and macrophage function. To elucidate the influence of SETIs to counter pro-tumorigenic conditions, at the early stages of HCC development, it was used as an established model of diethylnitrosamine/thioacetamide-injured liver fed with a standard diet (STD) or high-fat diet (42%) (HFD). The administration of SETIs improved survival and ameliorated tumor burden via modulation of monocyte-derived macrophages as key effectors involved in diet-induced HCC development. RT-qPCR analyses of hepatic tissue evidenced a diet-independent downregulatory effect of SETIs on the transcripts of CD36, FASN, ALOX15, and SREBP1c; however, animals fed with an STD showed opposing effects for PPAR and NRLP3 levels. These effects were accompanied by a decreased production of IL-6 and IL-17 but increased that of TNF in animals receiving SETIs. Moreover, only animals fed an HFD displayed increased concentrations of the stem cell factor. Overall, SETIs administration decreased the hepatic contents of lysophosphatydilcholine, phosphatidylinositol, phosphatidylcholine, and phosphatidyl ethanolamine. Notably, animals that received SETIs exhibited increased hepatic proportions of CD68^+^CX3CR1^+^CD74^+^ cells and at a higher rate in those animals fed an HFD. Altogether, the data evidence that oral administration of SETIs modulates the tumor microenvironment, improving hepatic innate immune response(s) and favoring a better antitumoral environment. It represents a path forward in developing coadjutant strategies to pharmacological therapies, with either a preventive or therapeutic character, to counter physiopathological conditions at early stages of HCC development.

## 1. Introduction

Hepatocellular carcinoma (HCC) is a major health problem with high morbidity and mortality, which often implies immune system disorders and local myeloid infiltration in hepatic tissue [1]. Macrophages, as a main type of innate immune effector, can play opposing roles in regulating the tumor microenvironment, cell progression, and severity of the disease according to its different phenotype subtypes. Various risk factors for HCC have been well-defined, including liver damage caused by inflammation and metabolic syndrome [2]. Other cofactors such as innate immune ‘Toll-like’ receptor (TLR)-4 activation and gut microbiota have also been involved in the control and promotion of HCC [3]. Over the last decade, there has been an improvement in the understanding of the genetic pathogenesis of HCC; however, only a few of the main drivers responsible for tumor initiation and progression have been identified as druggable targets [4]. Of note, recent data suggest that environmental inflammation-associated conditions such as non-alcoholic fatty liver disease (NAFLD) or the severe variant non-alcoholic steatohepatitis (NASH) precedes the development of HCC [5]. Improved knowledge of the relative contribution of environmental immunometabolic factors to the overall inflamed hepatic network could help to develop and/or establish more effective intervention strategies coadjutant to classical pharmacological approaches with a preventive or therapeutic character.

While the host’s endogenous factors influencing the risk of suffering HCC are difficult to influence, the environmental factors are predominant and addressable in a preventive or therapeutic intention. In this sense, recent discoveries have pointed to the close relationship between lipid homeostasis and metabolic derangements in liver cancer [6]. Thus, strategies to manipulate the tumor microenvironment in chemically injured livers of animals developing HCC are actively being investigated [7,8]. Our previous research has shown that *Chenopodium quinoa* seeds constitute a good source of serine-type protease inhibitors (SETIs) found in the globulin fraction, which display innate immune and metabolic effects ameliorating the HCC severity in mice [7,8,9,10]. The elucidation of functional and structural features showed that bioactive SETIs from *C. quinoa* appear as glycoproteins with a glucoside prosthetic group in complexes formed by homomeric subunits [9]. These compounds exhibited a significant partial resistance to gastrointestinal enzymes and, therefore, low bio-accessibility rates [10]. These compounds were mostly responsible for the effects showing biological correlation with TLR4 signaling [9,10]. SETIs appear to function primarily in the induction of a delayed wave downstream of TLR4 signaling [10]. Particularly, studies on high-fat diet (HFD) fed mice have shown beneficial innate immune and metabolic effects within the gut–liver axis targeting hepatocarcinogenesis derived from the administration of extracts enriched in serine-type protease inhibitors (SETIs) [8]. In this context, it was possible to target the hepatic lipid distribution of mono- and poly-unsaturated (PUFA) fatty acids that improved the hepatocellular susceptibility to ferroptosis associated with increased PUFA levels [8].

To the best of our knowledge, only studies on microglial priming by fatty acids have shown that HFD consumption increased the gene expression of markers such as CX3CR1, MHC-II, and NLRP3 [11]. Interestingly, these studies concluded the participation of an additional mediator, contributing to the exaggerated inflammatory response observed. When considering HCC development, it could be hypothesized that environmental modulation of innate immune responses via TLR4 activation enable disease modulation [3]. Additionally, the chemokine receptor CX3CR1 is expressed on various cells of the macrophage lineage [12]. This receptor modulates liver inflammation by upregulating pro-inflammatory cytokines such as tumor necrosis factor-α (TNF-α) and by favoring the infiltration of inflammatory monocytes [13]. The important roles of the NFκB-mediated CX3CL1/CX3CR1 axis and NLR family in liver injury are known [14]. In this context, recent research associated CD74^+^ (major histocompatibility complex II-associated invariant chain, MHC-II) macrophages with favorable prognosis and immune contexture in hepatocellular carcinoma [15]. Besides the recognized role of CD74 in antigen presentation, it also plays key roles in inflammatory diseases such as liver fibrosis [16,17], and macrophage inflammation and function [16,18]. Additionally, the activation of TLRs on macrophages results in the production of multiple soluble mediators including the key inflammatory cytokines TNFα and IL-6, which display opposing regulatory functions on the immune response via TLR4; whereas TNFα associates with TLR4 hypo-reactivity, IL-6 increases the responsiveness to activate NFκB [19]. Thus, as a consequence of environmental innate immune stimulatory conditions, subsets of monocyte-derived cells can generate a broad and diverse spectrum of innate immunometabolic responses, either worsening or improving HCC development and severity.

In view of the pivotal role that tumor microenvironment plays in the natural history of HCC, this study focuses on the potential immunomodulatory role of naturally occurring SETIs in modulating the pro-tumorigenic microenvironment at early stages of HCC development in metabolic models of diethylnitrosamine/thioacetamide (DEN/TAA)-injured livers.

## 2. Materials and Methods

### 2.1. Isolation of Protease Inhibitors (SETIs)

Innate immune and metabolically active extracts were obtained as described elsewhere [8]. In brief, commercial samples of *Chenopodium quinoa* seeds were purchased from local supermarkets (Madrid, Spain) to be finely grounded. The aliquots (0.5 g) were weighed into a centrifuge tube (50 mL) and extracted (×2) with 5 mL of phosphate-buffered saline solution (PBS) at 37 °C with gentle agitation during 2 h. The samples were thermal treated (60 °C) for 30 min, followed by sequential centrifugation: first (8000× *g*), to remove the precipitate and, second (10,000× *g*), to remove the supernatants using a 30 kDa membrane (Amicon^®,^ Merck KGaA, Darmstadt, Germany). Clear filtrates were filtered through 0.45 μm and subjected to reverse-phase liquid chromatography–ESI–MS analyses to confirm (i) the presence of glucoside-carrying proteins with adequate molecular weights and (ii) the absence of bacterial lipopolysaccharide (LPS) contamination [10]. Only samples fulfilling these conditions were lyophilized and kept frozen until use.

### 2.2. Induction of the Hepatocarcinoma (HCC)

C57Bl/6 male mice were obtained from the Centro de Investigaciones Biológicas (CIB-CSIC) (Madrid, Spain). To induce hepatocarcinoma (Ethic code, Proex 220/17), the animals were randomly distributed into four different groups (*n* = 6 per group) under a standard (groups 1 and 2) or a high-fat diet (groups 3 and 4) (AIN93G mod. HF 43 kcal% fat, irradiated, Ssniff spezialdiäten gmbh): groups 1 and 3 received a saline solution as the ‘vehicle’ (control); groups 2 and 4 received SETIs (reconstituted lyophilisates).

**Figure 1:**
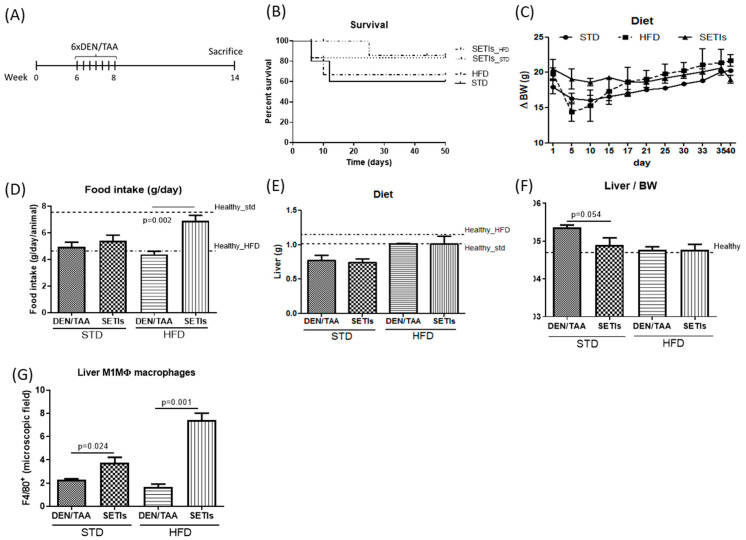
Liver tumor development in mice administered diethylnitrosamine (DEN) and the hepatotoxin thioacetamide (TAA). (**A**) Schematic representation of the procedure to induce the hepatocarcinoma in mice fed with a standard diet (STD) or a high-fat diet (HFD). (**B**) Survival curve (days) of serine-type protease inhibitors (SETIs) administered to mice fed with a standard diet (STD) or a high-fat diet (HFD). (**C**) Body weight gain of mice fed an STD or HFD, and those fed an HFD and administered SETIs were measured over 7 weeks. (**D**) Food intake in STD- or HFD-fed mice, receiving or not receiving SETIs, was measured every 48 h during the study period. (**E**) Liver masses in STD- or HFD-fed mice, receiving or not receiving SETIs, measured at week 7. (**F**) Liver to body weight (BW) ratio of DEN/TAA-treated mice fed with STPIs. (**G**) The number of infiltrated F4/80^+^ macrophages in hepatic tissues of STD- and HFD-fed mice receiving or not receiving SETIs was determined at week 7 after first DEN/TAA administration. Results are expressed as mean ± mean standard error (SEM) (*n* = 6).

HCC was induced by a combined treatment with diethynitrosamine (DEN)/thioacetamide (TAA) [3,7,8]: DEN (20 mg/kg, i.p.), given 3 times per week (for 2 weeks), and TAA (saturated solution—0.5 mL/kg i.g., dissolved in PBS—dose equivalent to 80 mg/kg) administered 3 times per week (for 8 weeks). The mice were sacrificed 7 weeks after the initial DEN injection (Figure 1).

Changes in body weight (BW) and food consumption were monitored every two days. After treatment, the mice were sacrificed by cervical luxation. Whole blood samples were preserved in EDTA-treated tubes (at room temperature) for analyses. Different sections (30–50 mg) of the liver were immersed in RNA later buffer (Qiagen, Hilden, Germany), Krebs’s buffer, and RIPA buffer and kept at −80 °C until analysis.

### 2.3. Quantitative Reverse Transcription Real-Time Polymerase Chain Reaction (qRT-PCR)

Validated Gene Expression Assays for murine (*Mus musculus*) CD36 (forward 5′-AAA AGC CAA GCC AGT GAC AAG-3′, reverse 5′-GGA CGC TTT TTC CTC AAG TCA-3′), fatty acid synthase (FASN) (forward 5′- TTC CCA CCA AGT GTG GGT AT -3′, reverse 5′- TGG GAC CTT CAG CTT GCT TC -3′), sterol regulatory element-binding protein 1 (SREBP1c) (forward 5′- TCA AAA CCG CTG TGT CCA GT -3′, reverse 5′- GAC GTC TCA ACC CGC TAG G -3′), arachidonate 15-lipoxygenase (ALOX15) (forward 5′- TCC CAT TCT AGG GGA GAG GG -3′, reverse 5′- CCT TGA CCA GCT CAG TAG GC -3′), peroxisome proliferator activated receptor (PPAR)-γ (forward 5′- TTG GTG GGA TTG TGT CTC GG -3′, reverse 5′- GGC CAA GAT CTC ACA GTG CT -3′), NRLP3 inflammasome (forward 5′- GGT GAC CAC GGG ACA AAA CT -3′, reverse 5′- ATT TGG TCC CAC ACA AGC -3′), and β-Actin (forward 5′-TGC ACC AAC TGC TTA-3′, reverse 5′-GGA TGC AGG GAT GAT GTT-3′) were purchased from Applied Biosystems (Foqter City, CA, USA).

PCR reactions were performed with 50 ng of cDNA from liver sections, using the Universal PCR Master Mix (Applied Biosystems). The cycling conditions were 10 min of denaturation at 95 °C and 40 cycles at 95 °C for 15 s and at 65 °C for 30 s and 72 °C for 20 s. Quantitative values were calculated by using the 2^−ΔCt^ method, wherein the ΔCt value of each sample was calculated by subtracting the average Ct value of the target gene from the average Ct value of the β-Actin gene.

### 2.4. Cytokine Production

Liver aliquots (25 mg) kept in Krebs’s buffer (0.3 mL) containing the Complete Protease Inhibitor Cocktail (Sigma, St. Louis, MO, USA) were thawed up and homogenized using a TissueRuptor (Qiagen) freshly before use. Then, the samples were centrifuged (10,000× *g*/15 min) to obtain clear supernatants for cytokine determination. Interleukin (IL)-6 (Cat. n° 32670069), IL-17 (Cat. n° 32670179), Tumor necrosis factor (TNF)-α (Cat. n° 32673019), and stem cell factor (SCF) (Cat. n° 32673329) were determined by ELISAs according to the manufacturer’s instructions (ImmunoTools, Friesoythe, Germany).

### 2.5. Analysis of the Hepatic Phospholipid Profile

Liver aliquots (50 mg) kept in Krebs’s buffer (0.2 mL) were homogenized. Whole homogenates were added (0.4 mL) with a 20:80 methanol/acetonitrile solution and extracted at 37 °C with gentle agitation during 1 h. The analyses were carried out on an Agilent HPLC system using a HILIC (75 × 3.2 mm) (Agilent) column. The elution phases consisted of mobile phase A (acetonitrile, 0.1% formic acid) and mobile phase B (deionized 18.2 MΩ cm water, 0.1% formic acid). Aliquots (30 µL) of the precipitation supernatants were injected in each cycle, and the analysis was performed with the following gradient (min, %B): 0, 5; 3, 5; 7, 50; 10, 50; 15, 100; 18, 100; 18.1, 5; and 20, 5.

Phospholipids were identified by matching the retention times and by co-injecting the samples with the phospholipid mixture for HPLC used as standards (P3817, Merck-Sigma, Darmstadt, Germany).

### 2.6. Immunohistochemistry for Hepatic F4/80^+^ Cells

The liver (4 μm) sections, prepared from tissue samples kept in O.C.T. [7], were incubated with a blocking solution (1X PBS/5% bovine serum/0.3% Triton™ X-100) for 60 min. After blocking, samples were washed with PBS. Fluorescent-tagged antibodies (F4/80) (Biolegend) were diluted in the dilution buffer (1X PBS/1% BSA/0.3% Triton™ X-100) and pipetted onto the slides. The samples were incubated overnight. Subsequently, after 3 washes of 10 min each in PBS-Triton X-100 0.05% (*v*/*v*), the liver sections were analyzed using an inverted fluorescence microscope Leica DM IL LED.

### 2.7. Immunophenotyping of Hepatic Infiltrated Cells

Liver aliquots (150 mg) kept in Krebs’s buffer (0.5 mL) were immersed in 0.5 mL of a trypsin/EDTA mixture (0.25%, sterile-filtered, 2.5 g porcine trypsin, and 0.2 g EDTA) (T4049, Merck-Sigma, Darmstadt, Germany). The samples were incubated at 37 °C with gentle shaking. Afterwards, large particles from tissue sections were completely disaggregated using pre-separation cell strainers (70 µm) (130-041-407 Miltenyi Biotech, Bergisch Gladbach, Germany).

After centrifugation (1200 rpm/5 min), the cells were resuspended in PBS and mixed with the following fluorochrome-conjugated antibodies: Anti-mouse CD68 (Cat # F-2761, Thermo-Fisher, Waltham, MA, USA), CX3CR1 (Cat No: 567805, BD Biosciences, Franklin Lakes, NJ, USA), CD74 (Cat No: 740939, BD Biosciences), and TLR4 (Cat No: 558294, BD Biosciences). Then, the samples were prepared for flow cytometry analysis with the Immunoprep kit (Beckman Coulter, Brea, CA, USA) according to the manufacturer’s instructions and further analyzed on a SORP Flow Cytometer (BD Biosciences).

### 2.8. Statistical Analyses

Statistical analyses were performed using SPSS v.15 software (SPSS Inc., Chicago, IL, USA). For normally distributed data, ANOVA with the post hoc Tukey test were applied, and for nonnormally distributed data from microbial analyses, the Mann–Whitney U test was used. Statistical significance was established at *p* < 0.05 for all comparisons.

## 3. Results

### 3.1. Amelioration of Tumor Burden in Hepatocarcinoma-Developing Mice

Major features in the development of HCC in mice fed a standard diet (STD) or high-fat diet (HFD) were measured (Figure 1). Cohorts of HCC-developing mice and age-match animals receiving SETIs were assessed for survival (Figure 1B). Animals administered SETIs displayed a significantly lower mortality in comparison with those only subjected to the concurrent administration of the DEN/TAA solutions. Herein, ≥80% of chemically treated mice completed the 7 weeks of treatment. This value represents a 20% higher survival percentage in comparison with the groups not receiving SETIs, regardless of the feeding diet. The sharp losses in body weight gain (∆BW) (Figure 1C) between the initial 10 days suggests severe inflammatory hepatitis as the main cause of liver failure and death. Notably, the administration of SETIs was able to ameliorate the curve at this early stage of treatment and could explain the higher survival percentages quantified. This effect may be interpreted as a preservation of the liver function as it was accompanied of an increased food intake in HFD-fed mice (Figure 1D), while STD-fed mice only showed an upward trend in food intake. None of the mice administered SETIs that completed the study period displayed significant changes in liver masses compared with those receiving the STD or HFD alone (Figure 1E). In addition, of the mice that reached the treatment, SETI-administered mice did not display a significantly higher liver/body weight ratio than their respective counterparts (Figure 1F). Both STD- or HFD-fed mice receiving SETIs displayed significantly a higher number of intrahepatic F4/80^+^ macrophages (Figure 1G), reflecting resistance to HCC development and sustained metabolism. These findings point to monocyte-derived macrophages as key effectors involved in the diet-induced control of HCC development.

### 3.2. Modulation of Neoplastic Markers

**Figure 2:**
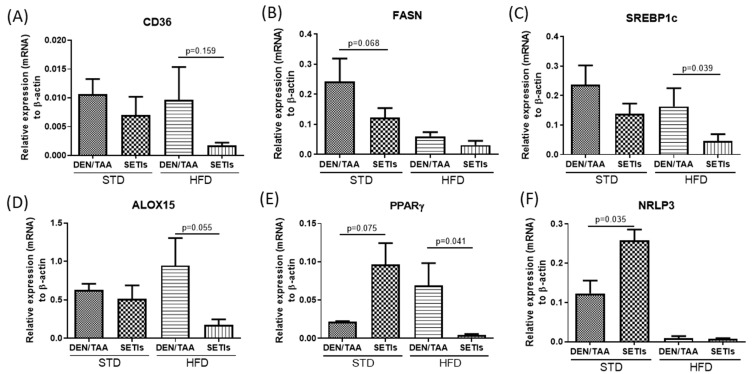
Interaction of SETIs with diet-induced lipid accumulation. Transcript levels (mRNA) of lipogenic markers: (**A**) CD36, fatty acid translocase; (**B**) FASN, fatty acid synthase; (**C**) SREBP1c sterol regulatory element-binding protein 1; (**D**) the coronary risk marker-arachidonate 15-lipoxygenase (ALOX15). Expression levels of the ‘double-edged’ neoplastic markers: (**E**) PPARγ, Peroxisome proliferator-activated receptor; (**F**) NRLP3 inflammasome. Results are expressed as mean ± mean standard error (SEM) (*n* = 4 for panels (**A**,**F**); *n* = 6 for panels (**B**–**D**)). Untreated controls are represented by the dotted line.

Rt-qPCR analyses of hepatic tissue revealed significant differences in the control of the selected markers depending on the diet fed to the groups of treatment (Figure 2). The administration of SETIs downregulated the expression of CD36 to a higher extent in animals fed an HFD (Figure 2A). In animals fed an HFD, a diet-induced significant decrease in the transcripts of FASN was quantified. Thus, the administration of SETIs only caused significant changes in the expression of FASN in the STD-fed group (Figure 2B). The data for SREBP1c and ALOX15 showed similar trends. For both markers, the administration of SETIs seemed to not influence the expression levels in the groups fed an STD, but the transcripts for both markers were significantly decreased in animals receiving SETIs that were fed an HFD. When considering the effects in the expression of PPARγ and NRLP3, the administration of SETIs sharply increased the expression levels for both makers in animals fed an STD, while significant opposite patterns were quantified in HFD-fed mice. Collectively, these data highlight the interference of molecular pathways triggered by SETIs with the lipid management and homeostasis. Data from SREBP1c and PPARγ could suggest a potential increased phagocytic condition in animals administered SETIs, which may interpret the results in a way to obtain better control of the lipid accumulation influencing innate immune activation to ameliorate HCC severity under an HFD.

### 3.3. Modulation of Inflammatory Mediators and Cellular Pattern Recognition Agonists

**Figure 3:**
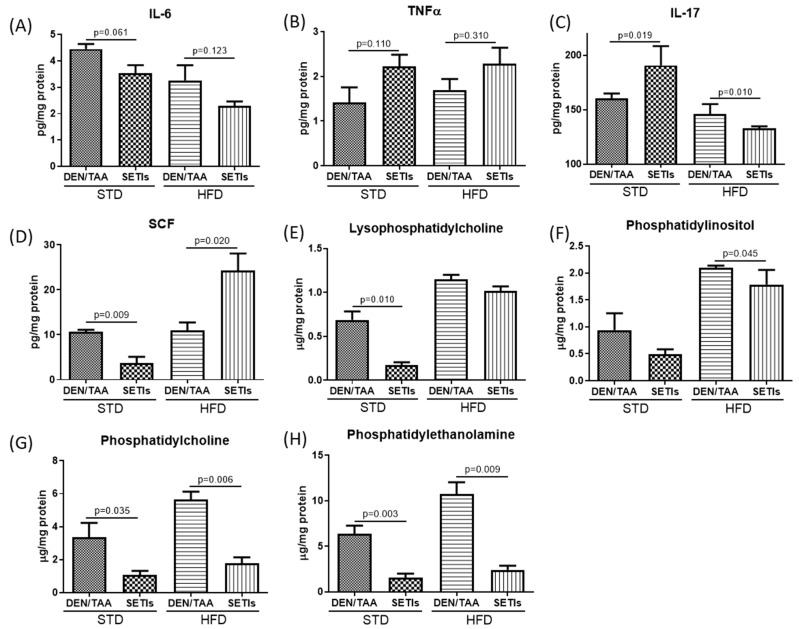
Hepatic concentration of immunological mediators in diethylnitrosamine (DEN)/thioacetamide (TAA)-treated mouse livers at 7 weeks, receiving or not receiving serine-type protease inhibitors (SETIs). Tissue cytokine concentration: (**A**) interleukin 6; (**B**) interleukin 17; (**C**) tumor necrosis factor (TNF)-α; (**D**) stem cell factor (SCF); and phospholipids (**E**) lysophosphatydilcholine—LPC, (**F**) phosphatidylinositol—PI, (**G**) phosphatidylcholine—PC, and (**H**) phosphatidylethanolamine—PE. Results are expressed as mean ± mean standard error (SEM) (*n* = 4 for panels (**A**–**D**); *n* = 6 for panels (**E**–**H**)).

The administration of SETIs led to a significant downregulation of IL-6 protein production (by 20%) in the groups fed an STD (Figure 3A), while this effect occurred to a higher extent (by 33%) in mice fed an HFD. However, an opposite trend was quantified for TNFα (Figure 3B), showing increased mean values of 57% and 34% in STD- and HFD-fed mice receiving SETIs, respectively. For both IL-6 and TNFα, variations in the same direction despite the diet fed to animals were quantified. Otherwise, contrasting patterns were quantified in the production of IL-17 and SCF depending on the diet (Figure 3C,D). Animals fed an STD receiving SETIs displayed increased levels of IL-17, associated with a decreased production of SCF, while feeding an HFD decreased the concentration of IL-17 but increased that of SCF. Altogether, SETI administration favors a cytokine profile in line with better controlled development of HCC. Decreased levels of cytokines mediating immunosuppression (i.e., IL-6), inhibition of autophagy, and promotion of cell migration (i.e., IL-17) together with the development of enriched granulocyte-macrophage colony-forming cells (i.e., SCF) may be interpreted as the experimental data showing immunostimulatory effects, which ameliorate HCC progression.

**Figure 4:**
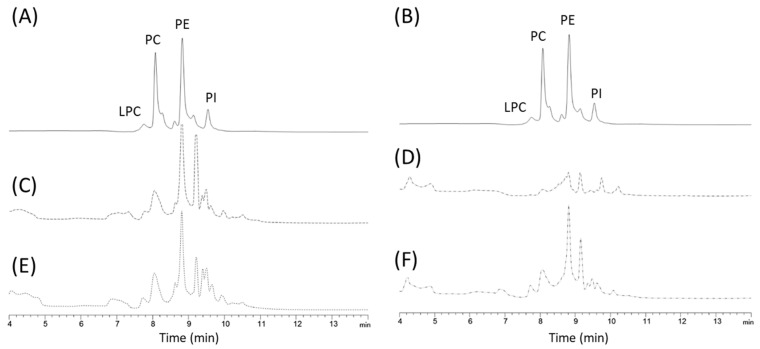
Overlaid HPLC-RP-UV chromatograms for the methanol/ACN extract of DEN/TAA-treated mouse livers at 7 weeks, receiving or not receiving SETIs. (**A**,**B**) Chromatographic signals for the standard mixture composed of Lysophosphatydilcholine (LPC), Phosphatidylinositol (PI), Phosphatidylcholine (PC), and Phosphatidylethanolamine (PE). (**C**,**D**): Chromatographic signals for hepatic extracts from mice fed a standard diet (STD) without receiving SETIs (**C**) or administered STEIs (**D**). (**E**,**F**) Chromatographic signals for the hepatic extracts from mice fed a high-fat diet (HFD) without receiving SETIs (**E**) or administered STEIs (**F**).

To investigate the effects of such cytokines on the levels of hepatic phospholipids (PLs), the levels of major PLs that play important roles in the development and progression of hepatocellular carcinoma were quantified (Figure 3). The chromatogram obtained for the methanol/ACN extract of DEN/TAA-treated mouse livers at 7 weeks, receiving or not receiving SETIs are shown in Figure 4. Feeding an HFD increased the hepatic content of PLs in animals not receiving SETIs. Moreover, mice administered SETIs, feeding either under an STD or HFD, exhibited downward trends or significant lower levels of the different PLs quantified. All PLs were significantly decreased in animals fed an STD. Otherwise, in animals fed an HFD, a slight negative variation of the LPC and PI concentration was quantified while PC and PE appeared to be significantly reduced. Overall, data demonstrate that PL metabolic pathways are coherently altered when animals are administered SETIs to impair hepatocyte switch to proliferation. Unfortunately, the PL speciation method does not allow us to identify oxidized PLs that could be recognized by soluble and cellular pattern recognition receptors (i.e., ‘Toll-like’ receptors) to modulate innate immunoreactivity.

### 3.4. Promotion of Infiltrated Macrophages with Antitumoral Activity

**Figure 5:**
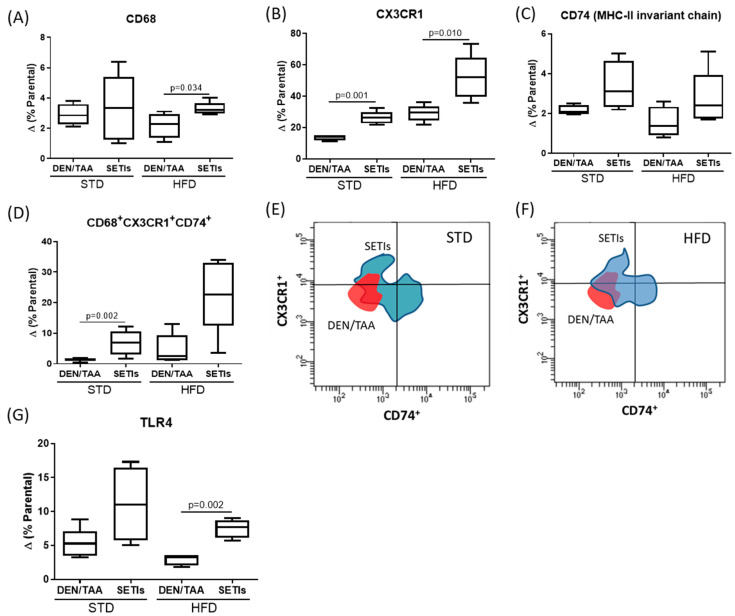
Immunophenotyping of the infiltrated macrophage population in DEN/TAA-treated mouse livers at 7 weeks, receiving or not receiving SETIs. Flow cytometric analysis of intrahepatic (**A**) CD68^+^ cells, (**B**) CD68^+^CX3CR1^+^ cells, (**C**) CD68^+^CD74^+^ cells, and (**D**) CD68^+^CX3CR1^+^CD74^+^ cells. Initially, the gating strategy was used to search the CD68^+^ population for double-positive CD68^+^TLR4^+^ cells. This region was further plotted to identify the double-positive cells for CX3CR1^+^ and CD74^+^: (**E**) overlaid density plots of the changes of the CX3CR1^+^CD74^+^ population in mice fed a high-fat diet (HFD) that received or did not receive serine-type protease inhibitors (SETIs); (**F**) overlaid density plots of the changes in the CX3CR1^+^CD74^+^ population in mice fed a standard diet (STD) that received or did not receive serine-type protease inhibitors (SETIs); and (**G**) CD68^+^CX3CR1^+^CD74^+^TLR4^+^ cells. Results are expressed as mean ± mean standard error (SEM) (*n* = 4 for panel (**G**); *n* = 6 for panels (**A**–**F**)).

Based on the above results, the promotion of hepatic infiltration by the monocyte-derived macrophage population (ihMθ) and its immunophenotyping in mice was directly examined (Figure 5). The ihMθ population was detected as CD68^+^CX3CR1^+^CD74^+^. Feeding animals with an HFD did not result in the infiltration of CD68^+^ macrophages into hepatic tissue, a condition linked to HCC development. When animals were administered SETIs, significantly increased proportions of CD68^+^ macrophages in the hepatic tissue of mice fed an HFD were observed, whereas STD-fed mice only display upward trends (Figure 5A). Given this finding and the association of M1 macrophages (Figure 1F) with antitumoral activity, CX3CR1 and CD74 identification in the infiltrated CD68^+^ population was performed. In HFD-fed animals, a significant effect directly caused by the diet was quantified. However, for both STD- and HFD-fed animals, the administration of SETIs significantly increased the CX3CR1^+^ population (Figure 5B). When considering CD74, proportions of the CD68^+^CD74^+^ population were similar among the two diets (Figure 5C). Only mice fed an STD displayed significant increases in the CD68^+^CD74^+^ population. The combination of all these markers allowed us to identify increased proportions of CD68^+^CX3CR1^+^CD74^+^ cells under either STD- or HFD-fed conditions (Figure 5D). Changes in the proportion of these populations are shown in the density plots (Figure 5E,F). Collectively, these data highlight that SETI administration is involved in the induction of antitumoral macrophage populations, allowing for a better control of the tumoral microenvironment. Increased levels of clusters of differentiation contributing to macrophage survival in tumor metastasis and their differentiation into pro-inflammatory F4/80^+^ macrophages in the liver (i.e., CX3CR1^+^CD74^+^) associated with a better survival rate allow for the results to be interpreted as supporting myeloid-derived antitumoral responses. All of these changes were associated with increased values for fluorescent staining of TLR4.

## 4. Discussion

In this study, the unexpected role of SETIs as innate immune agonists in the small intestine in the induction of hepatic immunity to ameliorate HCC severity is revealed. Mice either fed with an STD or HFD administered with SETIs, which are poorly digested as well as absorbed, were less prone to suffering the pathological consequences derived from HCC development. Previously, our own reports have shown that SETI administration is involved in the modulation of the innate immunity responses influencing HCC aggressiveness and progression. This effect was associated with increased proportions of F4/80^+^ cells in injured livers and innate immune effector cells (mRNA, CD68/CD206 ratio) in small intestinal tissue [7,8].

Analysis of the hepatic tissue from mice receiving SETIs points to the involvement of the monocyte-derived macrophage population in both lipid management and subsequent amelioration of HCC severity. SETI administration seems to play a critical role in activation in M1 macrophages, contributing to the development of an antitumoral immunophenotype. The potential role played by the monocyte-derived activated macrophage population (ihMθ) could be drawn out from the better preserved ∆BW gain during the early stages of HCC development (1–10 days). In fact, HFD-fed mice receiving SETIs showed significantly increased food intake compared with those fed an STD. Hepatocyte contribution to this effect could be excluded because food consumption was similar for both STD- and HFD-fed mice not receiving SETIs. However, along this line, previous research showed that human macrophage-like cells challenged by SETIs from *Chenopodium quinoa* displayed higher O_2_ consumption (by 33%) than controls [10]. The latter may reflect a sustained basal metabolism in SETI-administered mice. Similarly, because HFD-fed mice are as susceptible to HCC as STD-fed mice, it is likely that ihMθ contributes to modulating lipid management as the hepatic proportion of infiltrated F4/80^+^ cells and increase on food intake are higher in mice receiving SETIs. Intrahepatic accumulation of activated M1 macrophages can have important consequences preventing the host regulatory innate immunity from running out of control towards non-resolving inflammation. The latter is a well-accepted feature that markedly contributes to the development and progression of HCC [20].

Scavenger receptor CD36 is constitutively expressed in macrophages, in which the transcripts tend to be lowered in M1-polarized macrophages in response to hypoxia [21]. Moreover, CD36 plays a central regulatory role in NLRP3 inflammasome activation by facilitating non-resolving inflammation [22]. Here, the administration of SETIs appears to mediate intestinal innate immune activation, which coordinates hepatic immunometabolic events influencing NLRP3-inflammasome activation. The different effects observed in STD- or HFD-fed mice exhibit a direct association with the intrahepatic proportions of ihMθ. Notably, however, the inhibition of FASN and, therefore, the synthesis of long-chain fatty acids prevents TLR4-associated proinflammatory responses in macrophages [23]. These effects lay down the link between FASN and the cholesterol synthesis, which constitutes a major stimulus to orchestrate inflammasome activation [24]. The prevention of NRLP3 inflammasome in animals administered SETIs may contribute to its less prone activation in fibroblasts, which is further linked with progression and metastasis [25]. This assumption is supported by decreased transcripts of CD36, which promotes fibrogenic effects to remove apoptotic cells [26], and the better survival rates of mice receiving SETIs. Together, data appear to reveal a more substantial effect in HFD-fed mice in comparison with those under an STD that is clearly reflected in the fatty acid synthesis by SREBP1c. Accordingly, variations in the transcripts for ALOX15 transpire in the sense that SETIs promote a moderate antitumor role and function. The lower levels of ALOX15 support a better controlled and low level of hypoxia that favors a better-preserved hepatic basal metabolism in animals receiving SETIs in relation to their counterparts. These lipid-associated effects could explain, at least in part, the opposite changes in PPARγ expression. In STD-fed mice, SETI administration favors glucose metabolism to a higher extent, while in those under an HFD, the hepatic function and cellular differentiation may display a tighter control on glucose availability. Macrophage phenotype is highly associated with their metabolism, forcing oxidative metabolism in M1 macrophages to favor their polarization towards the M2 end [27].

The maturation of immune cell phenotypes and tissue function determine the production of diverse local inflammatory mediators that play key roles in HCC emergence, tumor microenvironment, progression, and severity. Indeed, SETI administration exerted a significant effect, decreasing the concentration of accepted immunosuppressive tumor markers for HCC such as IL-6 [28,29]. A positive correlation between IL-6 and the size of the tumor has been previously reported [30]. Accordingly, SETI administration was shown to be effective in reducing the number and size of the intrahepatic nodules of mononuclear cells (INMCs) in DEN/TAA-injured levels [7]. This effect appears to be independent of the enabled production of TNFα after SETI administration, which commonly occurs for IL-6 in obesity-promoted HCC development via the activation of oncogenic transcription factor STAT3 [31]. Notably, the TNFα levels could be directly associated with improved survival rates, which have been shown to display an inverse association with TNFα inhibition and deletion in a rat liver cancer model constructed by DEN treatment [32].

The positive effects on survival rates could also be explained, at least in part, by a potential interaction affecting TLR4-associated downstream molecular events. Signaling via TLR4/MyD88 initiates the activation of the IL23/IL17 axis that is involved in HCC cell proliferation and metastasis [33]. In vitro molecular studies showed that SETIs are engaged in downstream molecular signals related to TLR4 towards the production of IFNγ [10]. Moreover, studies on MyD88^−/−^ mice have associated those with increased frequency of splenic CD4^+^ T cells producing IL-10 and IL-17 [34], while IL-17 induces most of the same genes as TLR ligands [35]. Additionally, previous research efforts have showed that SETI administration influences iron-metabolism increasing bioactive hepcidin levels in the bloodstream [7,8] as well as the susceptibility to ferroptosis [8]. Here, these interactions appear to occur dependent on the phenotype of the ihMθ population in accordance with an increased production of IFNγ and iron sequestration in macrophages depleting the microenvironment, thereby limiting tumor growth while fostering inflammation. These synergistic interactions with the variation in SCF levels are associated not only with an increase in the number of macrophages but also with their selective differentiation towards an M1-like phenotype that develops in injured livers. Altogether, these processes have been associated with robustly predict clinical prognosis for HCC [36,37].

Macrophage polarization toward a classically activated (i.e., LPS/IFNγ) phenotype in inflammation has been demonstrated to occur via PL modulation [38,39,40]. The innate immune activation of macrophages results implies the substantial remodeling of arachidonate-containing phospholipids, leading to the mobilization of large quantities of this fatty acid for conversion into biologically active eicosanoids. After SETI administration, the decreased PL concentrations may interpret the results as a clear reflection of intense PL cleavage, allowing for further processing of SETIs. The latter could contribute to favoring a stabilized macrophage M1 phenotype [39] and their active implication in the regulation of innate immune responses [38]. Otherwise, PL incorporation into plasmalogens [41] could explain the diet-induced effect on the hepatic PL concentration and the moderate antitumor [42] role and function of SETIs. Collectively, data may suggest that SETI administration promotes innate immune activation, attenuating PL cleavage, to counter the development of liver disease, suggesting a possible mechanism of the HCC amelioration effect of SETIs during the early stages of HCC development. Overall, specific immunometabolic processes affecting lipid pathways are coherently altered to prevent survival and liver function.

FACS analysis confirmed that, after SETI administration, the ihMθ population acquired an M1-like phenotype (i.e., CD68^+^CX3CR1^+^CD74^+^). This migration pattern was consistent with the observation that CX3CR1, a chemokine receptor regulating differentiates F4/80^low^ monocytes into pro-inflammatory F4/80^high^ macrophages [43], was significantly expressed in both STD- and HFD-fed mice receiving SETIs. Meanwhile, the mean concentration of CD74, with favorable effects increasing the infiltration of CD8^+^ cytotoxic T lymphocytes in HCC [15], also allows us to hypothesize positive effects ameliorating the DEN/TAA-induced fibrotic consequences [17]. This phenotype highlights that the SETI-mediated activation of innate immunity drives recruitment and the selective functional differentiation of antitumor macrophages in HCC-developing mice. The fact that mice fed an HFD receiving SETIs displayed higher survival rates during HCC development also supports the lack of any consequence impairing the recruitment of ihMθ to the injured livers. These data point to diet-induced functional differences in TLR4 signaling during HCC development [3,44] between mice fed an STD or HFD. Interestingly, SETI administration contributed to maintaining a less prone macrophage population to immunosuppressive metabolic events in the tumor microenvironment [15,43].

## 5. Conclusions

Inflammatory and immunometabolic responses after serine-type protease inhibitor (SETI) administration to STD- or HFD-fed mice exert positive biological effects of these compounds during the early stages of hepatocarcinoma development. The extent to which a tumor inflammatory and metabolic microenvironment is influenced appears to be dependent on the type of diet. Particularly, SETIs play relevant roles in the phenotypic conversion and pro-inflammatory functional polarization of hepatic infiltrated macrophages. These effects are reflected in the promotion of a stabilized intrahepatic macrophage M1-like phenotype (CD68^+^CX3CR1^+^CD74^+^). Overall, SETI administration exerts a moderate antitumor role and function in a DEN/TAA mice model.

## Data Availability

The data that support the findings of this study are available from the corresponding author upon reasonable request.

## References

[1] Zhang Q., He Y., Luo N., Patel S.J., Han Y., Gao R., Modak M., Carotta S., Haslinger C., Kind D. (2019). Landscape and Dynamics of Single Immune Cells in Hepatocellular Carcinoma. Cell.

[2] European Association For The Study Of The Liver (2012). EASL–EORTC Clinical Practice Guidelines: Management of hepatocellular carcinoma. J. Hepatol..

[3] Dapito D.H., Mencin A., Gwak G.-Y., Pradere J.-P., Jang M.-K., Mederacke I., Caviglia J.M., Khiabanian H., Adeyemi A., Bataller R. (2012). Promotion of hepatocellular carcinoma by the intestinal microbiota and TLR4. Cancer Cell.

[4] Zucman-Rossi J., Villanueva A., Nault J.-C., Llovet J.M. (2015). Genetic Landscape and Biomarkers of Hepatocellular Carcinoma. Gastroenterology.

[5] Llovet J.M., Zucman-Rossi J., Pikarsky E., Sangro B., Schwartz M., Sherman M., Gores G. (2016). Hepatocellular carcinoma. Nat. Rev. Dis. Prim..

[6] Satriano L., Lewinska M., Rodrigues P.M., Banales J.M., Andersen J.B. (2019). Metabolic rearrangements in primary liver cancers: Cause and consequences. Nat. Rev. Gastroenterol. Hepatol..

[7] Laparra J.M., Fotschki B., Haros C. (2019). Immunonutritional consequences of different serine-type protease inhibitors in a C57BL/6 hepatocarcinoma model. Oncotarget.

[8] Laparra J.M., Brown D., Saiz B. (2020). Chenopodium Quinoa and Salvia Hispanica Provide Immunonutritional Agonists to Ameliorate Hepatocarcinoma Severity under a High-Fat Diet. Nutrients.

[9] Laparra J.M., Haros C.M. (2019). Plant seed protease inhibitors differentially affect innate immunity in a tumor microenvironment to control hepatocarcinoma. Food Funct..

[10] Srdić M., Ovčina I., Fotschki B., Haros C.M., Llopis J.M.L. (2020). *C. quinoa* and *S. hispanica* L. Seeds Provide Immunonutritional Agonists to Selectively Polarize Macrophages. Cells.

[11] Butler M.J., Cole R.M., Deems N.P., Belury M.A., Barrientos R.M. (2020). Fatty food, fatty acids, and microglial priming in the adult and aged hippocampus and amygdala. Brain. Behav. Immun..

[12] Yona S., Kim K.-W., Wolf Y., Mildner A., Varol D., Breker M., Strauss-Ayali D., Viukov S., Guilliams M., Misharin A. (2013). Fate mapping reveals origins and dynamics of monocytes and tissue macrophages under homeostasis. Immunity.

[13] Karlmark K.R., Zimmermann H.W., Roderburg C., Gassler N., Wasmuth H.E., Luedde T., Trautwein C., Tacke F. (2010). The fractalkine receptor CX3CR1 protects against liver fibrosis by controlling differentiation and survival of infiltrating hepatic monocytes. Hepatology.

[14] Ren J., Meng S., Yan B., Yu J., Liu J. (2016). Protectin D1 reduces concanavalin A-induced liver injury by inhibiting NF-κB-mediated CX3CL1/CX3CR1 axis and NLR family, pyrin domain containing 3 inflammasome activation. Mol. Med. Rep..

[15] Xiao N., Li K., Zhu X., Xu B., Liu X., Lei M., Sun H.-C. (2021). CD74+ macrophages are associated with favorable prognosis and immune contexture in hepatocellular carcinoma. Cancer Immunol. Immunother..

[16] Su H., Na N., Zhang X., Zhao Y. (2017). The biological function and significance of CD74 in immune diseases. Inflamm. Res..

[17] Heinrichs D., Knauel M., Offermanns C., Berres M.-L., Nellen A., Leng L., Schmitz P., Bucala R., Trautwein C., Weber C. (2011). Macrophage migration inhibitory factor (MIF) exerts antifibrotic effects in experimental liver fibrosis via CD74. Proc. Natl. Acad. Sci. USA.

[18] Heinrichs D., Berres M.-L., Coeuru M., Knauel M., Nellen A., Fischer P., Philippeit C., Bucala R., Trautwein C., Wasmuth H.E. (2014). Protective role of macrophage migration inhibitory factor in nonalcoholic steatohepatitis. FASEB J..

[19] Tamandl D., Bahrami M., Wessner B., Weigel G., Ploder M., Fürst W., Roth E., Boltz-Nitulescu G., Spittler A. (2003). Modulation of Toll-Like Receptor 4 Expression on Human Monocytes by Tumor Necrosis Factor and Interleukin-6: Tumor Necrosis Factor Evokes Lipopolysaccharide Hyporesponsiveness, Whereas Interleukin-6 Enhances Lipopolysaccharide Activity. Shock.

[20] Yu L.-X., Ling Y., Wang H.-Y. (2018). Role of nonresolving inflammation in hepatocellular carcinoma development and progression. NPJ Precis. Oncol..

[21] Raggi F., Pelassa S., Pierobon D., Penco F., Gattorno M., Novelli F., Eva A., Varesio L., Giovarelli M., Bosco M.C. (2017). Regulation of Human Macrophage M1–M2 Polarization Balance by Hypoxia and the Triggering Receptor Expressed on Myeloid Cells-1. Front. Immunol..

[22] Sheedy F.J., Grebe A., Rayner K.J., Kalantari P., Ramkhelawon B., Carpenter S.B., Becker C.E., Ediriweera H.N., Mullick A.E., Golenbock D.T. (2013). CD36 coordinates NLRP3 inflammasome activation by facilitating intracellular nucleation of soluble ligands into particulate ligands in sterile inflammation. Nat. Immunol..

[23] Carroll R.G., Zasłona Z., Galván-Peña S., Koppe E.L., Sévin D.C., Angiari S., Triantafilou M., Triantafilou K., Modis L.K., O’Neill L.A. (2018). An unexpected link between fatty acid synthase and cholesterol synthesis in proinflammatory macrophage activation. J. Biol. Chem..

[24] Guo C., Chi Z., Jiang D., Xu T., Yu W., Wang Z., Chen S., Zhang L., Liu Q., Guo X. (2018). Cholesterol Homeostatic Regulator SCAP-SREBP2 Integrates NLRP3 Inflammasome Activation and Cholesterol Biosynthetic Signaling in Macrophages. Immunity.

[25] Ershaid N., Sharon Y., Doron H., Raz Y., Shani O., Cohen N., Monteran L., Leider-Trejo L., Ben-Shmuel A., Yassin M. (2019). NLRP3 inflammasome in fibroblasts links tissue damage with inflammation in breast cancer progression and metastasis. Nat. Commun..

[26] Pennathur S., Pasichnyk K., Bahrami N.M., Zeng L., Febbraio M., Yamaguchi I., Okamura D.M. (2015). The macrophage phagocytic receptor CD36 promotes fibrogenic pathways on removal of apoptotic cells during chronic kidney injury. Am. J. Pathol..

[27] Rodríguez-Prados J.-C., Través P.G., Cuenca J., Rico D., Aragonés J., Martín-Sanz P., Cascante M., Boscá L. (2010). Substrate fate in activated macrophages: A comparison between innate, classic, and alternative activation. J. Immunol..

[28] Porta C., De Amici M., Quaglini S., Paglino C., Tagliani F., Boncimino A., Moratti R., Corazza G.R. (2008). Circulating interleukin-6 as a tumor marker for hepatocellular carcinoma. Ann. Oncol. Off. J. Eur. Soc. Med. Oncol..

[29] Chen Y.-Q., Li P.-C., Pan N., Gao R., Wen Z.-F., Zhang T.-Y., Huang F., Wu F.-Y., Ou X.-L., Zhang J.-P. (2019). Tumor-released autophagosomes induces CD4+T cell-mediated immunosuppression via a TLR2–IL-6 cascade. J. Immunother. Cancer.

[30] Malaguarnera M., Di Fazio I., Laurino A., Romeo M.A., Giugno I., Trovato B.A. (1996). Role of interleukin 6 in hepatocellular carcinoma. Bull. Cancer.

[31] Park E.J., Lee J.H., Yu G.-Y., He G., Ali S.R., Holzer R.G., Osterreicher C.H., Takahashi H., Karin M. (2010). Dietary and genetic obesity promote liver inflammation and tumorigenesis by enhancing IL-6 and TNF expression. Cell.

[32] Jing Y., Sun K., Liu W., Sheng D., Zhao S., Gao L., Wei L. (2018). Tumor necrosis factor-α promotes hepatocellular carcinogenesis through the activation of hepatic progenitor cells. Cancer Lett..

[33] Kang Y., Su G., Sun J., Zhang Y. (2018). Activation of the TLR4/MyD88 signaling pathway contributes to the development of human hepatocellular carcinoma via upregulation of IL-23 and IL-17A. Oncol. Lett..

[34] Kader M., Alaoui-El-Azher M., Kode B., McArthur M., Shinde A., Wells A., Ismail N. (2016). MyD88 suppresses IL-10 and IL-17 production in response to obligate intracellular *Ehrlichia* infection. J. Immunol..

[35] Shen F., Ruddy M.J., Plamondon P., Gaffen S.L. (2005). Cytokines link osteoblasts and inflammation: Microarray analysis of interleukin-17- and TNF-α-induced genes in bone cells. J. Leukoc. Biol..

[36] Zhang J.-P., Yan J., Xu J., Pang X.-H., Chen M.-S., Li L., Wu C., Li S.-P., Zheng L. (2009). Increased intratumoral IL-17-producing cells correlate with poor survival in hepatocellular carcinoma patients. J. Hepatol..

[37] Tang B., Zhu J., Li J., Fan K., Gao Y., Cheng S., Kong C., Zheng L., Wu F., Weng Q. (2020). The ferroptosis and iron-metabolism signature robustly predicts clinical diagnosis, prognosis and immune microenvironment for hepatocellular carcinoma. Cell Commun. Signal..

[38] Gil-de-Gómez L., Astudillo A.M., Meana C., Rubio J.M., Guijas C., Balboa M.A., Balsinde J. (2013). A Phosphatidylinositol Species Acutely Generated by Activated Macrophages Regulates Innate Immune Responses. J. Immunol..

[39] Qin X., Qiu C., Zhao L. (2014). Lysophosphatidylcholine perpetuates macrophage polarization toward classically activated phenotype in inflammation. Cell. Immunol..

[40] Barbosa C.M.V., Bincoletto C., Barros C.C., Ferreira A.T., Paredes-Gamero E.J. (2014). PLCγ2 and PKC Are Important to Myeloid Lineage Commitment Triggered by M-SCF and G-CSF. J. Cell. Biochem..

[41] Ismail I.T., Elfert A., Helal M., Salama I., El-Said H., Fiehn O. (2021). Remodeling Lipids in the Transition from Chronic Liver Disease to Hepatocellular Carcinoma. Cancers.

[42] Zhan Y., Wang L., Liu J., Ma K., Liu C., Zhang Y., Zou W. (2013). Choline Plasmalogens Isolated from Swine Liver Inhibit Hepatoma Cell Proliferation Associated with Caveolin-1/Akt Signaling. PLoS ONE.

[43] Lee Y.-S., Kim M.-H., Yi H.-S., Kim S.Y., Kim H.-H., Kim J.H., Yeon J.E., Byun K.S., Byun J.-S., Jeong W.-I. (2018). CX3CR1 differentiates F4/80low monocytes into pro-inflammatory F4/80high macrophages in the liver. Sci. Rep..

[44] Kang H.H., Kim I., Lee H., Joo H., Lim J.U., Lee J., Lee S., Moon H. (2017). Chronic intermittent hypoxia induces liver fibrosis in mice with diet-induced obesity via TLR4/MyD88/MAPK/NF-kB signaling pathways. Biochem. Biophys. Res. Commun..

